# Iodol: An Effective Substitute for Iodoform

**Published:** 1887-09

**Authors:** 


### *IODOL: AN EFFECTIVE SUBSTITUTE FOR IODOFORM.*

*Practitioner*, Vol. XXXVIII., No. 5, 1887.

Dr. R. Norris Wolfenden, the author, concludes as follows: Iodol is very rich in iodine, containing only seven per cent. less than iodoform, but parting with it more readily than the latter body. No toxic symptoms follow its constant use, and it is, therefore, preferable to iodoform on this account, and also because it is quite as effective, and further possesses neither smell nor taste. It is one of the best applications for ulcerations of the mouth, pharynx, larynx, and nose, ozæna, scrofulous ulcerations, specific conditions, etc. The author confirms the statements of Lublinski, that the ulcers of phthisical laryngitis will heal completely under daily insufflations of iodol. He uses the following preparations:

1. Insufflations of pure powder. It is more important to cover the diseased surface than to measure the dose.

2. Mazzoni's solution: Iodol, 1 part; alcohol, 16 parts; glycerine, 34 parts—a useful brush application or coarse spray.

3. Iodol, 1 drachm; glycerine, 1 drachm; vaseline, 7 drachms—as a brush application.

4. Pastilles of iodol, iodol, 1 grain; glycerine, 1 minim; glycogelatine, 18 grains. These are much preferable to iodoform pastilles and are the most serviceable of all for chronic pharyngeal conditions.

5. Iodol, 1 drachm; ether, 1 ounce—a spray or brush application.

6. Iodol bougies, containing ½ grain iodol in each, for nasal conditions.

7. Iodol wool, 10 per cent. for tampons, etc.

8. Iodol gauze for dressings.

Iodol possesses all the properties of iodoform, is antiseptic, anæsthetic, a promotor of granulation and healing, arrests suppuration, and deodorizes foul secretions, and is to be preferred to iodoform on account of its pleasant and slight odor and absence of taste. Its effects are quite as rapidly obtained.—*Journal of Laryngology and Rhinology.*

